# Surgical Strategies and Clinical Outcome of Large to Giant Sphenoid Wing Meningiomas: A Case Series Study

**DOI:** 10.3390/brainsci10120957

**Published:** 2020-12-09

**Authors:** Adrian Balasa, Corina Hurghis, Flaviu Tamas, Rares Chinezu

**Affiliations:** 1 Department of Neurosurgery, George Emil Palade University of Medicine, Pharmacy, Science and Technology, 540142 Tîrgu Mureș, Romania; adrian.balasa@yahoo.fr (A.B.); hurghis.corina@gmail.com (C.H.); flaviu_tamas1989@yahoo.com (F.T.); 2 Department of Neurosurgery, Tîrgu Mureș Emergency Clinical County Hospital, 540136 Tîrgu Mureș, Romania

**Keywords:** sphenoid wing meningiomas, cavernous sinus, optic canal, vascular encasement, gross total resection, skull base invasion

## Abstract

Large to giant sphenoid wing meningiomas (SWMs) remain surgically challenging due to frequent vascular encasement and a tendency for tumoral invasion of the cavernous sinus and optic canal. We aimed to study the quality of resection, postoperative clinical evolution, and recurrence rate of large SWMs. This retrospective study enrolled 21 patients who underwent surgery between January 2014 and December 2019 for SWMs > 5 cm in diameter (average 6.3 cm). Tumor association with cerebral edema, extension into the cavernous sinus or optic canal, degree of encasement of the major intracranial arteries, and tumor resection grade were recorded. Cognitive decline was the most common symptom (65% of patients), followed by visual decline (52%). Infiltration of the cavernous sinus and optical canal were identified in five and six patients, respectively. Varying degrees of arterial encasement were seen. Gross total resection was achieved in 67% of patients. Long-term follow-up revealed improvement in 17 patients (81%), deterioration in two patients (9.5%), and one death (4.7%) directly related to the surgical procedure. Seven patients displayed postoperative tumor progression and two required reintervention 3 years post initial surgery. Tumor size, vascular encasement, and skull base invasion mean that, despite technological advancements, surgical results are dependent on surgical strategy and skill. Appropriate microsurgical techniques can adequately solve arterial encasement but tumor progression remains an issue.

## 1. Introduction

Meningiomas are the most common benign intracranial tumor, representing up to 18% of all intracranial tumor pathologies. Among the supratentorial locations, sphenoid wing meningiomas (SWMs) account for up to one-quarter [1,2,3,4,5,6,7,8].

Typically, meningiomas are slow growing and over 90% are benign lesions (World Health Organization grade 1) that do not infiltrate surrounding structures. As such, symptomatology onset is insidious and, consequently, the clinical presentation, surgical risk, and prognosis can vary from case to case [1,3,9,10,11,12,13].

**Figure 1:**
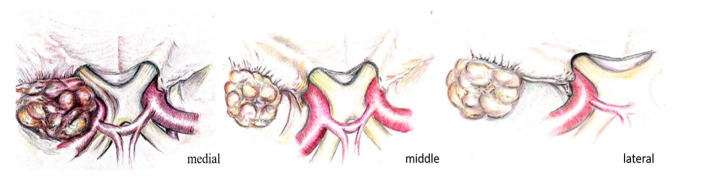
Meningiomas: localization along the sphenoid ridge.

Meningiomas originating along the sphenoidal ridge were first classified by Cushing and Eisenhardt as medial, middle, and lateral SWMs (Figure 1) [14].

Large to giant SWMs represent a surgical challenge not only because they come into contact with important anatomical structures such as the large intracerebral anterior circulation arteries, cavernous sinus, optic chiasm, and optic nerves, but also because they show a high recurrence rate, often infiltrating the bony structures [15,16,17,18,19,20,21].

Due to the rarity of this subcategory of SWMs, there are few studies addressing surgical results. There is no consensus in recent studies in regarding the optimal surgical solution, alternating from aggressive to conservative surgical attitudes, especially when dealing with significant skull base and vascular involvement [4,22,23,24,25,26,27,28,29,30].

The aim of our study was to evaluate the surgical results of large to giant SWMs, especially how the arterial encasement and cavernous sinus infiltration influences the postoperative clinical outcome, tumor recurrence rate, and its origin.

## 2. Materials and Methods

This is a retrospective case series study involving 21 consecutive patients with large to giant SWMs who underwent surgery in the Department of Neurosurgery, Tîrgu Mureș Clinical Emergency Hospital, Romania, between January 2014 and December 2019. All cases were histopathologically confirmed.

All subjects gave their informed consent for inclusion before they participated in the study. The study was conducted in accordance with the Declaration of Helsinki, and the protocol was approved by the Ethics Committee of our University.

### 2.1. Inclusion and Exclusion Criteria

We defined large to giant SWMs as tumors measuring greater than 5 cm in diameter in at least one direction. All patients operated on for the first time for large to giants SWMs were included in this study.

We considered en plaque meningiomas, cavernous sinus meningiomas, anterior clinoid meningiomas, and extensions of petroclival meningiomas to represent different pathologies, and as such, patients with these categories of tumor were excluded.

### 2.2. Clinical and Radiological Evaluation

Each patient was clinically evaluated at multiple timepoints: prior to surgical intervention, 24 h postoperatively, upon discharge, 3 months postoperatively, and annually thereafter. Clinical data were recorded in individual evaluation sheets that accompanied the patients throughout the monitoring period.

Cognitive function was evaluated using the Montreal Cognitive Assessment Test [31], visual function was evaluated using visual acuity and visual field tests, motor function was evaluated using the Medical Research Council scale [32], and aphasia was assessed using the Kaplan score [33].

**Figure 2:**
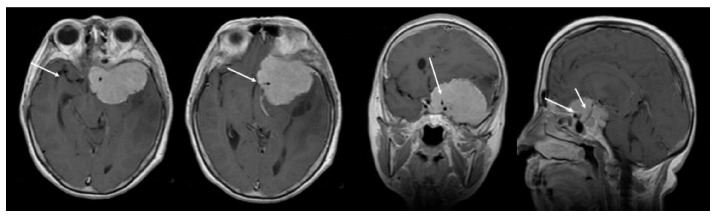
Total arterial encasement—internal carotid artery (ICA)—white arrow.

**Figure 3:**
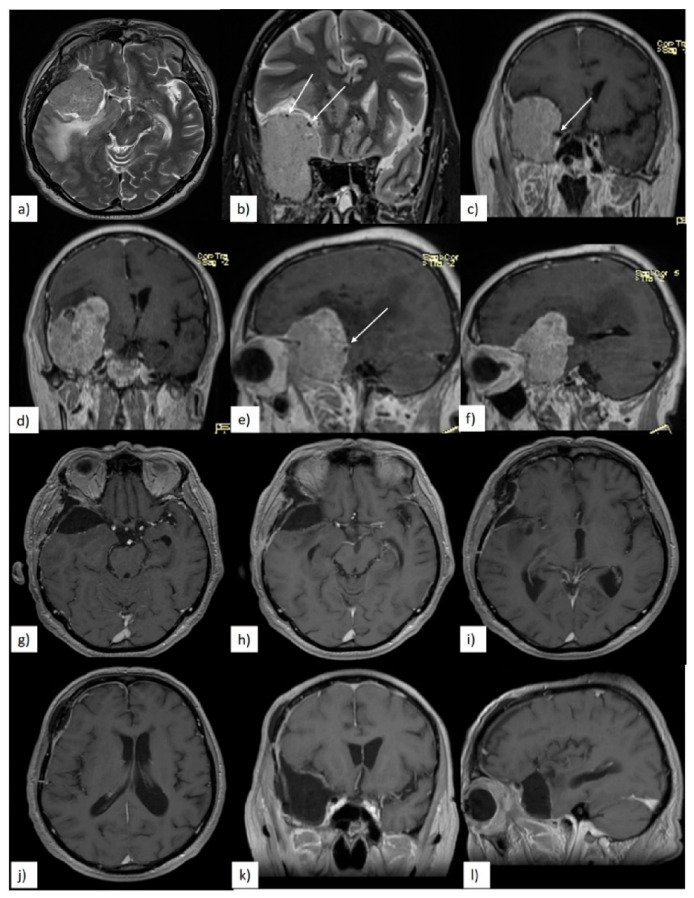
(**a**–**f**) Preoperative magnetic resonance imaging (MRI) showing partial arterial encasement of ICA- and middle cerebral artery (MCA)-white arrows; (**g**–**l**) 3-month postoperative T1 contrast MRI showing gross total resection (GTR) of the tumor.

Preoperative radiological evaluation was performed using a 1.5 Tesla magnetic resonance imaging (MRI) machine (T1- and T2-weighted sequences), with a focus on identifying tumor association with cerebral edema, tumor extension into the cavernous sinus or optic canal, and the degree of encasement of the major intracranial arteries. Arterial encasement was established as either total or partial. We defined total encasement as occurring when at least one of the major intracranial arteries was totally engulfed by tumoral tissue, and partial encasement when at least 50% of the arterial circumference was encased by tumoral tissue (Figure 2 and Figure 3).

### 2.3. Classification of Meningiomas

Although SWMs do not closely respect any classification system, especially in the case of large lesions, we used the original Cushing and Eisenhardt classification system [14], identifying each meningioma as either a lateral, middle, or medial SWM, according to the position of the tumoral insertion along the sphenoidal ridge (Figure 1).

### 2.4. Description of Surgical Technique

In the modern neurosurgical era, the aim of surgical intervention is gross total resection (GTR) of the tumor, with preservation or improvement of preoperative neurological status. All patients in this study were operated on using the pterional approach. To avoid brain retraction and spatula use, especially when the entire surface of the tumor was covered by brain tissue, a lumbar drain was used. This helped to achieve proper brain relaxation and thus facilitated preparation of the operative corridor.

**Figure 4:**
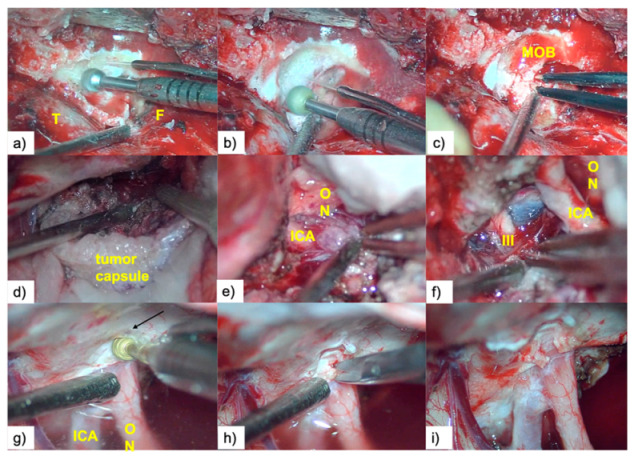
(**a**,**b**) Drilling the sphenoid ridge; (**c**) meningo-orbital band (MOB) was then coagulated and cut; (**d**) sphenoid wing meningioma (SWM) dissection using the dynamic retraction technique. We used the tumoral capsule as a “retractor”; (**e**) use of the suction canula to resect the tumor surrounding the left ICA; (**f**) oculomotor nerve (III) dissection and Liliequist membrane in the background; (**g**–**i**) optic canal unroofing and tumor resection from the optic canal.

Following removal of the bone flap, we proceeded with varying degrees of extradural drilling of the sphenoidal ridge, including, in selected cases, anterior clinoidectomy. This maneuver allowed for early devascularization of the tumor, safer tumor resection, and simultaneously, a significantly reduced surgical time (Figure 4a–c).

After opening the dura mater, dissection began with the wide opening of the distal sylvian fissure followed by careful sharp dissection of the sylvian veins and tracking of the distal branches of the middle cerebral artery (MCA) until they reached the posterior surface of the tumor. In order to reduce injury to an already compromised brain, we avoided the use of fixed retractors, instead applying the “dynamic retraction technique,” using the space already created by the tumor and its capsule as an “anatomical retractor” (Figure 4d).

Upon reaching the lateral surface of the tumor, we alternated internal debulking with progressive detachment of the tumor from its dural insertion along the sphenoidal ridge. Even in very fibrous tumors, intensive internal debulking allowed us to obtain a soft and malleable tumor capsule, facilitating further peritumoral neurovascular dissection.

**Figure 5:**
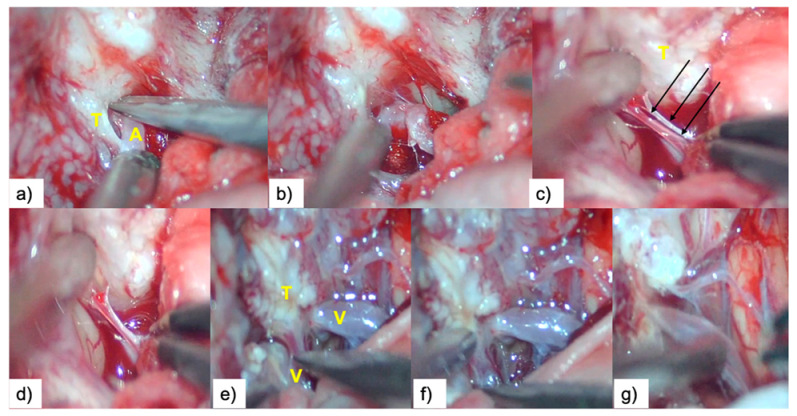
Vascular dissection: (**a**,**b**) arterial (A) dissection; (**c**,**d**) perforating arteries dissection (arrows); (**e**–**g**) venous (V) dissection. T = tumor.

This was followed by retrograde sharp arterial venous dissection in a distal to proximal direction, paying attention to preservation of the perforating arteries. Our surgical strategy was to leave a small fragment of tumoral tissue attached to the artery in cases with very fibrous tumors, rather than seeking aggressive periarterial dissection that increases the risk of damaging the arterial wall; (Figure 5a–g).

Once the optic nerve was identified, the dissection plane was followed, preserving the microvasculature of the optic pathways using a sucker with teardrop-shaped suction control (which permits regulation of suction intensity) as a dissector, and avoiding intensive use of bipolar coagulation. If the tumor extended into the optic canal, early decompression of the proximal optic nerve by wide unroofing of the optic canal was performed (Figure 4g–i).

### 2.5. Resection Quality

The senior author and an independent radiologist scored resection quality using Simpson [34] grading, based on the operative report coupled with examination of the postoperative contrast-enhanced T1-weighted MRI images. We categorized resections with a Simpson grade of II or III as GTR, and those with a Simpson grade of IV as subtotal resection (STR).

## 3. Results

### 3.1. Patient Population

Between January 2014 and December 2019, 83 patients diagnosed with SWMs underwent surgery in the Neurosurgery Department of the Emergency Clinical Hospital in Tîrgu Mureș, Romania. Of these, 21 patients (25%) met the inclusion criteria for this study. Average patient age was 57 (SD ± 12.37) years and the ratio of women to men was 1.33.

### 3.2. Preoperative Clinical and Radiological Data

Cognitive decline was the most frequent presenting symptom, occurring in 13 patients (62.0%). Of these, six patients (46.0%) presented with mild cognitive decline, five patients (38.5%) presented with a medium degree of cognitive decline, and two patients (15.5%) presented with severe cognitive decline. Preoperative visual dysfunction was present in 11 patients (52.0%). Of these, six patients (54.5%) presented with a visual field deficit and five patients (45.5%) presented with an ipsilateral decrease in visual acuity (Table 1).

Nine patients (43.0%) presented with a headache, six (28.5%) presented with varying degrees of contralateral motor deficit and aphasia, three (14.0%) presented with oculomotor nerve palsy, and two patients (9.5%) presented with seizures (Table 1).

In nine patients (43.0%), the tumor origin was in the medial region of the sphenoidal ridge, in six patients (28.5%) the origin was in both the middle and medial regions of the sphenoidal ridge, and in the remaining six patients (28.5%) the origin was in the lateral region of the sphenoidal ridge.

All tumors were associated with varying degrees of brain edema and mass effect.

Tumor infiltration of the cavernous sinus and the optic canal was identified in five patients (24.0%) and six patients (28.5%), respectively.

Major intracranial arterial engulfment was present in all patients; total encasement of at least one major artery (the internal carotid artery—ICA, MCA, or anterior cerebral artery) in 11 patients (52.0%), and partial encasement in the remaining patients (48.0%, *n* = 10) (Table 1).

The average tumor diameter was 6.3 cm, with a range of 5.0–7.4 cm.

### 3.3. Quality of Resection

All operations were performed in a single session, with GTR achieved in 14 patients (67.0%) and STR achieved in the remaining seven patients (33.0%).

**Figure 6:**
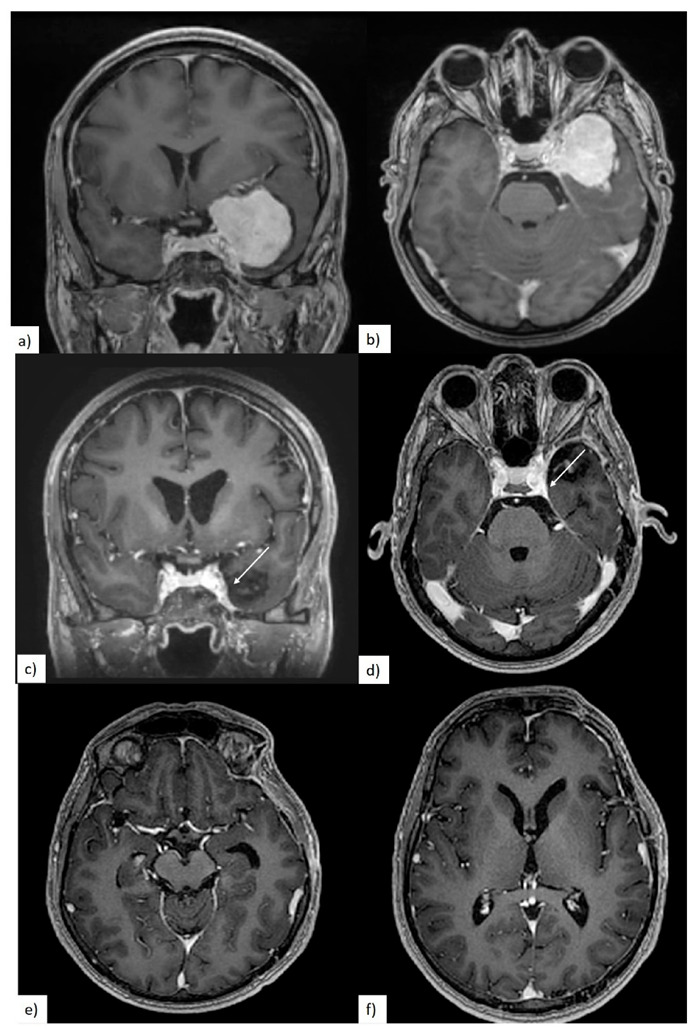
(**a**,**b**) Preoperative MRI showing cavernous sinus invasion; (**c**–**f**) postoperative MRI showing intracavernous remnant—white arrow.

In four out of five cases with cavernous sinus involvement, the surgeon reported that some residual tumor was intentionally left in place. In the remaining case, with a very soft meningioma, complete tumor resection from the cavernous sinus was achieved (Figure 6).

In two patients (9.5%), we identified residual tumors at the level of the ICA/MCA wall and in one patient (4.7%) a residual tumor was present at the level of the sphenoidal ridge on postoperative T1-weighted contrast-enhanced MRI. However, over 80.0% resection of the initial tumor was achieved in all STR cases. In all six patients with optic canal penetration, we succeeded in decompressing the optic apparatus, either directly or by unroofing the optic canal (Table 2).

### 3.4. Postoperative Clinical Evolution and Follow-up

The mean long-term follow-up period was 35 months, with a range of 10–60 months (excluding the early deaths of two patients).

In the immediate postoperative period, 14 patients remained neurologically stable and the neurological deficits of seven patients had worsened. Among them, preoperative motor deficits had degraded in two patients, one had transitory aphasia, and four patients displayed visual decline. However, 2 weeks after the surgery, four patients had at least recovered their preoperative neurological status, while three patients (two with visual disturbances and one with postoperative venous infarction) never recovered (Table 2). At the 3-month follow-up, all except three patients had a significantly improved neurological status.

Mortality in the series was 9.5% (*n* = 2). Despite aggressive therapy, including decompressive craniectomy, one patient died on the sixth postoperative day due to postoperative cerebral venous infarction and severe brain edema. The second patient died 3 months after surgery, due to a pulmonary thromboembolism.

In seven patients (33.0%), control MRI demonstrated tumoral progression. The origin of regrowth was the cavernous sinus in four cases, residual tumor adherent to the arterial walls in two cases, and tumoral residue located along the sphenoid ridge in one case. Three years after initial surgery, two patients with residual tumors originating at the level of the cavernous sinus required reintervention due to tumoral progression.

## 4. Discussion

SWMs represent a surgical challenge even in the modern neurosurgical era, especially in the case of giant lesions. In our study, large to giant SWMs represented 25% of the entire SWM series at our hospital. This high percentage signifies late diagnosis, most likely due to the relative tolerance of the brain to lesions located in this area, although interestingly we observed that the giant SWMs in our study were associated with different degrees of cerebral edema.

Anterior circulation arterial encasement was ubiquitous in our series, being present to varying degrees in all patients. Total arterial encasement was present in the majority of patients (52.0%) and the remaining patients had at least one major artery of the anterior circulation partially encased at the periphery of the tumor. Previous studies have revealed total arterial encasement to be present in almost 100% of cases, especially at the level of the supraclinoid ICA [3]. However, in our case series, 28.5% of meningiomas originated from the lateral portion of the sphenoidal wing, which may explain why total arterial encasement was present in around half of our patients.

In accordance with the findings of Kattner et al., we could not find any association between preoperative imaging and the degree of intraoperative adherence between the tumor and the major intracranial arteries [35].

Unsurprisingly, because of the notable proportion of SWMs originating from the medial and middle-medial portions of the sphenoidal wing (71.5%), tumor extension was directed towards the cavernous sinus and the optic canal in over 50% of cases, making surgery in these circumstances extremely challenging.

Compared to the findings of other studies that note visual deficits and headaches as the most frequently encountered symptoms [15,20,36], the majority of our patients presented with cognitive decline (62%). We assume cognitive decline was so prevalent because varying degrees of associated brain reaction were present in all our cases. Ipsilateral visual dysfunction was present in only 52% of our patients despite most SWMs being located in the medial or middle-medial portions of the sphenoidal wing. We believe this low percentage is a consequence of the severe cognitive decline experienced by many patients, who were unable to fully cooperate during visual testing.

The first resection of an SWM was performed in 1938 by Cushing and Eisenhardt [14]. Since then, different approaches have been described for appropriately exposing the sphenoidal wing [16,20,37,38,39,40,41]. In this case series, all patients were operated on using case-specific customizations of the pterional approach, depending on tumor extent. Although described in the literature as an alternative, we did not consider using the fronto-temporo-orbito-zygomatic approach as we consider this to be time consuming and excessively invasive [16,38,39,41]. We believe that the pterional approach, with appropriate use of the volume and space already created by the tumor, allows for an adequate and safe operative field.

It is well known that these lesions have a tendency to encase cerebral arteries and simultaneously extend into hard-to-reach areas (e.g., the cavernous sinus, optic canal, and orbit). These characteristics make surgery of giant SWMs extremely challenging, with significant differences in the GTR rates obtained [2,37,38,39,40,41,42].

Mustafa et al. explored the relationship between tumor dimension and resection degree, achieving a GTR rate of 95.2% for tumors measuring less than 4 cm in diameter and 58.6% for those over 4 cm in diameter [18]. Our GTR rate was slightly higher, at 67.0%, despite an average tumor diameter of 6.3 cm, which we believe indicates a high-level surgical technique. STR was achieved in seven patients (33.0%). Of these, residual tumor was noted at the level of the cavernous sinus in four patients (evident in postoperative MRI in two cases, and reported by the surgeon in two cases), and the surgeon reported residual tumor adherent to the main arterial axis or along the sphenoidal ridge in three patients. However, over 80% resection of the initial tumor was achieved in all STR cases in this series.

In two patients, the surgical report and the postoperative MRI evaluation revealed tumoral residue to be in contact with the major anterior circulation arteries. Nevertheless, we concluded that with adequate intratumoral debulking and careful use of sharp dissection, proper arterial dissection from the tumor capsule could be achieved in most situations without significant risks.

Several studies portray encased arterial dissection as the most risky procedure for surgical treatment of SWMs, and some propose that thin segments of tumor capsule should be left on the cerebral arteries [2,15,18,20,43,44]. McCracken et al. discuss the relationship between the degree of arterial encasement, the amount of tumor resected from the involved artery, and postoperative ischemia. They describe total encasement of the supraclinoid internal carotid artery, M1 segment, and A1 segment as being “the deadly triad”, recommending STR in this situation [44]. In this case series, when perforating arteries were encased by tumoral tissue, we avoided the use of bipolar coagulation, and by using a suction canula and microscissors we performed adequate dissection of the cleavage plane.

In our opinion, the most difficult aspect of SWM resection is the dissection and preservation of the sylvian veins. This is supported by the fact that the only death in this case series that was related to the surgical intervention was due to cerebral venous infarction, not an ischemic lesion. We consider the key factors in venous dissection to be the limitation of perivenous bipolar coagulation, thus avoiding compromising the arachnoid plane, and the use of sharp dissection away from the friable venous wall.

Cavernous sinus tumoral extension is the main factor influencing the degree of GTR of SWMs. Tumoral extension into the cavernous sinus can be approached using the different triangles described in the literature, although there are conflicting results regarding the preservation of the cranial nerves that cross this anatomical structure [2,45,46,47]. Nakamura et al. achieved total resection in 14.5% of cases when the cavernous sinus had been infiltrated by the tumor, compared to 92.3% when it had not. Similarly, the recurrence risk increased from 7.7% when the cavernous sinus had not been invaded, to 27.7% when it had been [41].

When tumor tissue extended into the cavernous sinus, our surgical strategy was a conservatory one—to preserve the function of the cranial nerves. However, in one patient with a soft meningioma, we were able to resect the intracavernous extension of the tumor by the use of soft suction, achieving complete tumoral resection.

In five cases, extensive unroofing of the optic canal was necessary. This maneuver not only allowed the resection of the intracanalar extension of the tumor, but also increased the mobility of the fixed optic nerve. All surgery around the optic apparatus was performed with careful preservation of the microcirculation of the optic pathways.

SWM surgery is considered very challenging, and is associated with many risks and postoperative complications [15,16,19,21]. Nevertheless, mortality rates have decreased in the last three decades thanks to advances both in neuroimaging and microsurgical techniques. Today, the range of reported mortality rates is 0–43% [18,23,26,27,29]. Our immediate postoperative mortality rate was 4.7%, excluding the patient that died 3 months after surgery because of a pulmonary thromboembolism.

In our series, seven patients (33.0%) developed immediate neurosurgical complications directly related to the surgical procedure. The most frequently encountered complication was visual deterioration, identified in four out of 11 patients with a preoperative visual deficit; two (18.0%) of these patients never recovered to the preoperative level of visual deficit. Champagne et al. reported an immediate postoperative complication rate of 25%, represented by one case of mixed aphasia and two cases of hemiparesis. However, the Champagne et al. study included only 12 cases [3].

All seven patients with postoperative tumoral residue demonstrated progression, assessed by MRI during the follow-up period, although only two patients (9.5%) with residual tumor inside the cavernous sinus required surgical reintervention for tumor extension outside of the original cavernous sinus remnant, which challenges the neurosurgical literature claiming that leaving small amounts of tumoral residue has little impact on tumor regrowth [3,48]. One interesting finding is that postoperative tumoral progression was mainly seen in cases where the residue was at the level of the cavernous sinus or along the sphenoidal ridge, and only one case (4.7%) showed progression from tumoral residue adherent to the MCA. We could speculate that periarterial tumoral residue presents a smaller risk of regrowth compared to skull base tumoral residue; however, further studies performed on a larger series of cases are necessary.

Our study is limited by the number of cases and the moderate period of follow-up. In order to evaluate long-term evolution and benefits of adjuvant radiotherapy, further multicentric studies are required.

## 5. Conclusions

An optimal surgical solution for giant SWMs is not yet readily accessible, and results are largely dependent on surgical strategy and skill. Appropriate microsurgical techniques can adequately solve arterial encasement but tumor progression remains an issue. This is especially true in patients with tumor invasion of the cavernous sinus, where the risk of tumoral progression is higher, prompting the need for continuous surveillance of these patients [49]. Larger multicenter studies and longer follow-up surveillance are needed to better understand the evolution of this pathology.

## Figures and Tables

**Table 1:** Preoperative clinical and radiological data.

Preoperative Clinical Data			Preoperative Radiological Evaluation		
Clinical Signs		*n* (%)	MRI Characteristics		*n* (%)
*cognitive decline*	mild	6 (46%)	*sphenoidal ridge tumor origin*	medial	9 (43%)
	medium	5 (38.5%)		middle and medial	6 (28.5%)
	severe	2 (15.5%)		lateral	6 (28.5%)
*visual disfunctions*	visual field	6 (54.5)	*cavernous sinus invasion*		5 (24%)
	visual acuity	5 (45.5)	*optic canal invasion*		6 (28.5%)
*headaches*		9 (43%)	*major arterial encasement*	total	11 (52%)
*aphasia*		6 (28.5)		partial	10 (48%)
*motor deficits*		6 (28.5)	*cerebral edema*		21 (100%)
*oculomotor nerve palsy*		3 (14%)	*average diameter*	6.3 cm ranging between 5–7.4 cm	
*seizures*		2 (9.5%)			

**Table 2:** Immediate postoperative and long-term follow-up.

Immediate Postoperative Clinical Evolution			Quality of Resection and Tumoral Progression		
Clinical Data		*n* (%)	Radiological Data		*n* (%)
*unchanged*		14 (67%)	*GTR*		14 (67%)
*worsened*	total	7 (33%)			
	visual disfunctions	4 (19%)	*STR*		7 (33%)
	motor deficits	2 (9.5%)	*location of residual tumor*	total	7
	aphasia	1 (4.7%)		cavernous sinus	4 (57%)
**long term clinical follow-up**				ICA/MCA	2 (29%)
*improved*		17 (81%)		sphenoidal ridge	1 (14%)
*worsened*		2 (9.5%)	*site of residual tumor which required surgical reintervention*	cavernous sinus	2 (9.5%)
*deaths*		2 (9.5%)			
*surgical reintervention*		2 (9.5%)			
